# Transmitted/founder SHIV.D replicates in the brain, causes neuropathogenesis, and persists on combination antiretroviral therapy in rhesus macaques

**DOI:** 10.1186/s12977-023-00628-5

**Published:** 2023-08-10

**Authors:** Rachel M. Podgorski, Jake A. Robinson, Mandy D. Smith, Suvadip Mallick, Huaqing Zhao, Ronald S. Veazey, Dennis L. Kolson, Katharine J. Bar, Tricia H. Burdo

**Affiliations:** 1 Center for NeuroVirology and Gene Editing, Department of Microbiology, Immunology, and Inflammation, Lewis Katz School of Medicine, Temple University, Philadelphia, PA USA; 2 Department of Medicine, Perelman School of Medicine, University of Pennsylvania, Philadelphia, PA USA; 3 Center for Biostatistics and Epidemiology, Department of Biomedical Education and Data Science, Lewis Katz School of Medicine, Temple University, Philadelphia, PA USA; 4 Tulane National Primate Research Center, Tulane School of Medicine, Covington, LA USA; 5 Department of Neurology, Perelman School of Medicine, University of Pennsylvania, Philadelphia, PA USA

**Keywords:** SHIV, Non-human primates, HIV, Persistence, NeuroHIV

## Abstract

A biologically relevant non-human primate (NHP) model of HIV persistence in the central nervous system (CNS) is necessary. Most current NHP/SIV models of HIV infection fail to recapitulate viral persistence in the CNS without encephalitis or fail to employ viruses that authentically represent the ongoing HIV-1 pandemic. Here, we demonstrate viral replication in the brain and neuropathogenesis after combination antiretroviral therapy (ART) in rhesus macaques (RMs) using novel macrophage-tropic transmitted/founder (TF) simian-human immunodeficiency virus SHIV.D.191,859 (SHIV.D). Quantitative immunohistochemistry (IHC) and DNA/RNAscope in situ hybridization (ISH) were performed on three brain regions from six SHIV.D-infected RMs; two necropsied while viremic, two during analytical treatment interruptions, and two on suppressive ART. We demonstrated myeloid-mediated neuroinflammation, viral replication, and proviral DNA in the brain in all animals. These results demonstrate that TF SHIV.D models native HIV-1 CNS replication, pathogenesis, and persistence on ART in rhesus macaques.

**Supplementary Information:**

The online version contains supplementary material available at 10.1186/s12977-023-00628-5.

## Introduction

Research has demonstrated that long-lived myeloid cell types play a critical role in human immunodeficiency virus-1 (HIV-1) latency and cellular dysfunction even during antiretroviral therapy (ART) suppression [1–4]. However, HIV persistence and reservoirs in the central nervous system (CNS) remain understudied. Simian immunodeficiency virus (SIV)/simian-human immunodeficiency virus (SHIV) infection of non-human primates (NHP) is a biologically relevant model to investigate HIV-1 persistence in the CNS, but the rapid pathogenic progression of previous SIV and SHIV viruses has limitations in modeling HIV-1 [5, 6]. Earlier studies utilizing SHIV/NHP model systems in the CNS were focused on SHIV/HIV encephalitis and did not investigate the persistent effects of SHIV infection in the CNS through ART suppression [7–11]. Furthermore, earlier models did not include TF HIV-1 envelope-containing viruses. To better understand the chronic progression of HIV-1 neuropathogenesis on suppressive ART, a non-encephalitic CNS infection NHP model is necessary [12]. Here, we characterize the neuropathogenesis of macrophage-tropic TF SHIV infection in rhesus macaques (RMs) to elucidate the dynamics of HIV pathogenesis and persistence in the brain during ART.

During suppressive ART therapy, stably integrated and replication-competent HIV-1 proviral DNA persists in several long-lived cell types [1, 4, 13–15]. If treatment is interrupted, HIV can rebound from latent reservoirs to detectable levels of viremia in as little as two weeks [16]. CD4 + memory T cells were the first HIV-1 reservoir identified and are the best characterized to date [13]. However, a myeloid-based viral reservoir in the CNS has been extensively described [17–21]. It is hypothesized that HIV-1 seeding of the CNS is caused by the transmigration of macrophages, CD14 + CD16 + monocytes, and CD4 + T cells across the blood-brain barrier [17, 20, 22, 23]. Recent studies have provided evidence that CNS macrophages and microglia are susceptible to HIV-1 infection and harbor HIV-1 DNA during ART-induced viral suppression [18–20, 24]. Due to the scarcity of human brain samples from ART-suppressed individuals and the neuropathological differences between traditional SIV/NHP models and HIV-1 neuropathogenesis, the viral dynamics and complete characterization of the latent reservoir in the CNS have yet to be fully elucidated. Biologically relevant, neuropathogenic TF SHIVs are a valuable tool to investigate the myeloid reservoir in the CNS *in vivo.*

Env SHIVs are chimeric viruses in which an HIV-1 env gene replaces the equivalent gene in an SIV backbone and thus encode HIV-1 *tat, rev, vpu*, and *env* genes [25]. SIVmac-backboned SHIVs encoding HIV-1 *env* glycoproteins efficiently replicate and cause disease in RMs [25]. In humans, productive HIV-1 infection in a naïve host is typically established by a single TF virus that founds productive systemic infection [26]. SHIV.D.191,859 is a TF virus identified from an acutely infected Ugandan woman in 2008. The isolated clade D HIV-1 virus is CCR5-tropic, with the ability to replicate in both CD4 + T cells and macrophages [27]. SHIV.D.191,859 encodes this HIV-1 Env within a SIVmac766 backbone, with the substitution of a bulky, hydrophobic residue at Env position 375, part of the CD4 binding domain, to enhance binding to rhesus CD4 molecules and replication in RM cells [28]. SHIV.D.191,859 recapitulates many features of HIV-1 pathogenesis, including mucosal and intravenous transmission, consistent viral kinetics, and CD4 depletion over time [25, 28]. Historically, infection with clade D HIV is associated with more severe neuropathogenesis and higher prevalence of HIV-associated neurocognitive decline than clades B, C and A, all of which have published information on their relationship to HIV-associated neurocognitive disorders [29, 30]. The desirable viral kinetics and macrophage tropism of this SHIV, and the reported neuropathogenesis of clade D HIV-1 [26, 27], provide unique opportunities for TF SHIV.D.191,859 as a neuropathogenic NHP model.

Previous research evaluated SHIV.D.191,859 for its potential to model HIV-1 for latency in the periphery [31]. Six RMs were infected with SHIV.D, treated with suppressive ART (tenofovir DF, emtricitabine, dolutegravir) for 24 weeks, and subjected to analytical treatment interruption (ATI) [31]. Quantification of cellular viral RNA (caRNA) and cell-associated proviral DNA (caDNA) from PBMCs during chronic infection and at several post-ART timepoints revealed stable levels of caDNA pre- and post-ART, as well as caRNA, rebound to pre-ART levels after ATI [31]. CD4 + T cell quantification revealed depletion in unsuppressed animals [31]. In comparison to SIVmac239 infection of RMs, the lower levels of viremia during peak and early infection characteristic of SHIV.D infection better models HIV-1 in humans [31]. The vast majority of SHIV.D-infected RMs achieved viral suppression with ART in two to four weeks [31]. This is advantageous over chronic SIVmac239 infection, which often takes many months to achieve suppression on ART [31]. Importantly, SHIV.D maintains CCR5 tropism, which is associated with the progression of AIDS-like disease in RMs [31, 32]. Here, we investigated the effects of TF SHIV.D.191,859 infection in the brain of RMs.

## Results

### SHIV.D infection in rhesus macaques

Six female RMs were infected with SHIV.D intravaginally or intravenously as previously described (Supplemental Table 1) [31]. A viral set point of > 10^3^ viral copies/mL (c/mL) was established for at least 24 weeks. ART (daily, subcutaneous emtricitabine, tenofovir disoproxil fumarate, and dolutegravir) was administered for 24 weeks, followed by ATI. Two RMs (FR55, GA67) were restarted on ART following ATI. Based on the ART status, immunopathogenesis, and viremia, the RMs were divided into three groups: (1) progression (n = 2), where RMs experienced plasma viremia > 10^6^ c/mL and were euthanized for clinical deterioration, (2) off ART (n = 2), where RMs underwent necropsy off ART without overt signs or symptoms of simian acquired immunodeficiency syndrome (AIDS), and (3) on ART (n = 2), where both RMs were necropsied on ART with suppressed viremia without signs or symptoms of simian AIDS. CNS and peripheral pathological findings at necropsy were recorded and scored. Three RMs had notable CNS pathological findings at the time of necropsy. EJ94 (progression) exhibited vacuolar degeneration and vacuolar lesions in the occipital lobe. FE43 (off ART) had small inflammatory foci in the occipital lobe, subcortical white matter, and meninges. FT42 (off ART) had perivascular inflammation in the frontal cortex, temporal lobe, and thalamus (Supplemental Table 1).

### SHIV.D infection is associated with inflammatory myeloid cells in the CNS

**Fig. 1:**
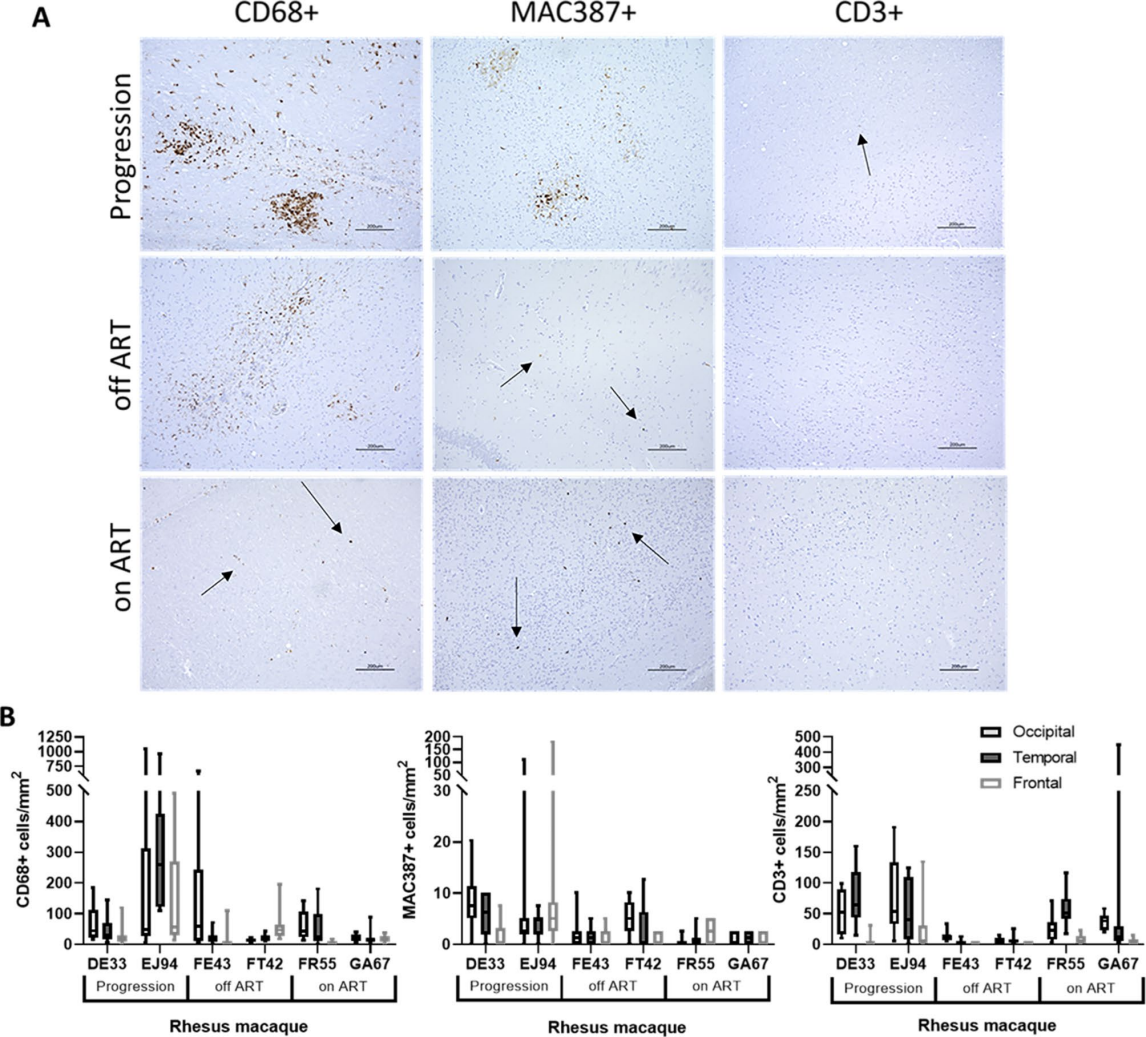
Quantitative immunohistochemistry of immune cell markers in TF SHIV.D RM brain tissue. Immunohistochemistry (IHC) staining of CD68+, MAC387+, and CD3 + cells in brain tissue of progression, off-ART, and ART suppressed RMs **(A).** Representative images from occipital, temporal, and frontal lobes at 10X magnification are shown. Scale bar = 200 μm. Target cells were quantified using Keyence BZ-X700 Microscope and accompanying Batch Analysis Software to determine the average number of IHC stained cells from 10 nonoverlapping 20X images per brain section of each RM **(B).** 20X frame area = 393,880 μm². Box and whisker plots are presented as mean values of counts ± quartiles, with whiskers representing the range

To characterize the degree of inflammation in the brain of the SHIV.D infected RMs, occipital, temporal, and frontal cortex sections were immunostained with antibodies against CD68 for resident tissue macrophages, MAC387 for recently infiltrated monocyte-derived macrophages, and CD3 for T lymphocytes and expression of each was quantified (Fig. 1A). Inflammatory lesions containing CD68 + and MAC387 + cells were seen in RMs from the progression group, and an accumulation of CD68 + macrophages was found in the off-ART group. Vacuolar lesions containing CD68 + macrophages and multinucleated giant cells were observed in Progression RM. Infiltration of MAC387 + monocyte-derived macrophages continued in the animals in the on-ART group. However, small inflammatory foci and perivascular inflammation persisted in the CNS of off-ART and on-ART RMs, including FT42, a spontaneous controller of plasma viremia. The highest levels of all quantified immune cell markers were found in the progression group (Fig. 1B). The mean number of CD68 + cells in the progression group was 46.6 (DE33) and 230.6 cells/mm^2^ (EJ94); in the off-ART group, 65.4 (FE43) and 31.1 cells/mm^2^ (FT42) and in the on-ART group, 39.7 (FR55) and 19.6 cells/mm^2^ (GA67). The mean number of MAC387 + cells in the progression group was 5.33 (DE33) and 13.3 cells/mm^2^ (EJ94). In the off-ART group, there were 1.52 (FE43) and 3.13 MAC387 + cells/mm^2^ (FT42), and in the on-ART group, 1.27 (FR55) and 1.02 MAC387 + cells/mm^2^ (GA67). The mean number of CD3 + T cells in the progression group was 44.7 (DE33) and 50.9 cells/mm^2^ (EJ94). In the off-ART group, there were 5.60 (FE43) and 4.31 CD3 + cells/mm^2^ (FT42). In the on-ART group, the mean was 31.0 (FR55) and 33.3 CD3 + cells/mm^2^ (GA67).

### Viral RNA and DNA persist in the CNS after 6 months ART

**Fig. 2:**
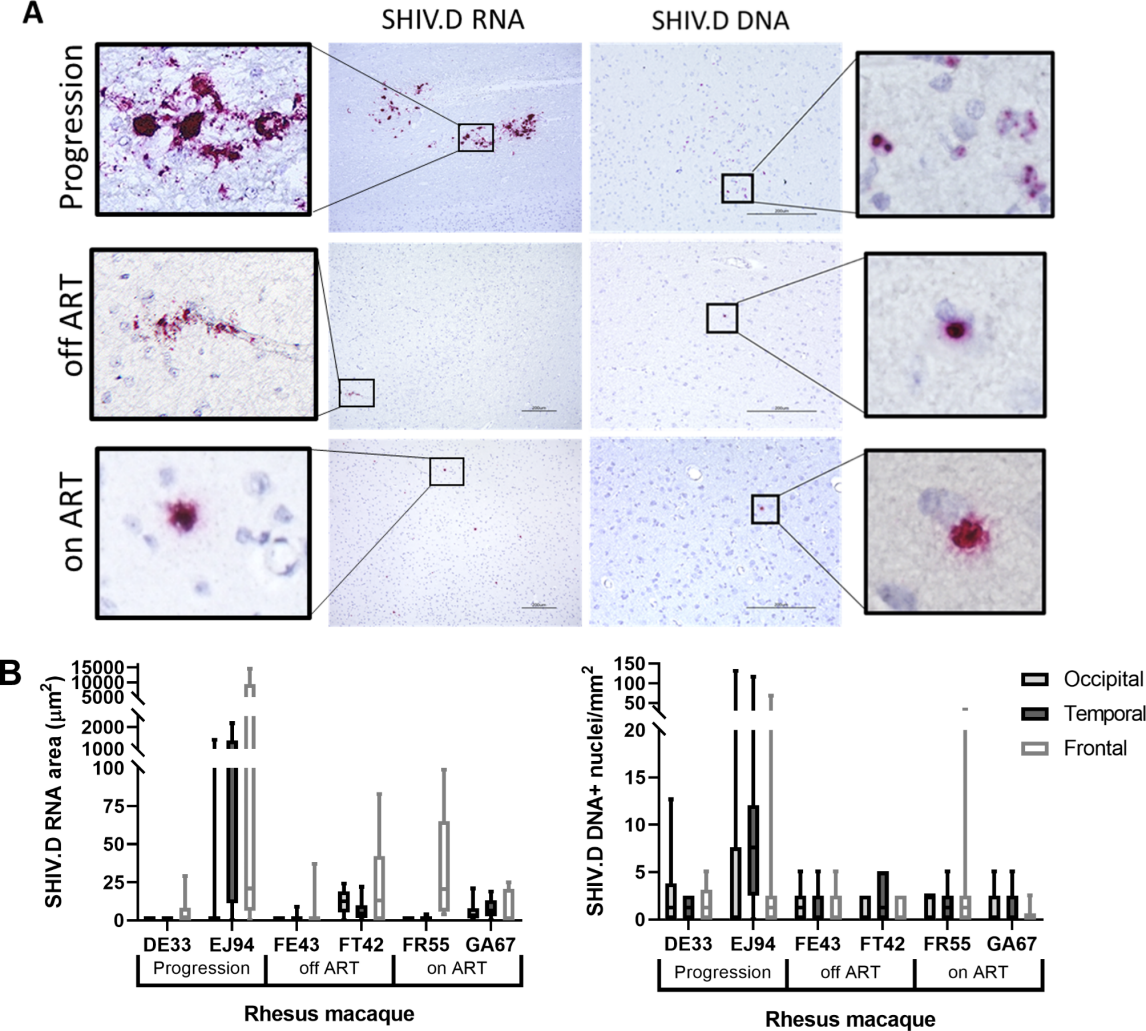
Quantitative viral RNA and DNA in situ hybridization in TF SHIV.D RM brain tissue. RNAscope (left) and DNAscope (right) in situ hybridization (ISH) of replicating viral RNA and proviral DNA in brain tissue of progression, off-ART, and ART suppressed RM **(A)**. Representative images from occipital, temporal, and frontal lobes are shown at 10X (RNA) and 20X (DNA) magnification. Inserts = 40X magnification. Scale bar = 200 μm. The area of SHIV.D RNA positive signal (µm^2^) or the number of nuclei containing proviral DNA/mm^2^ were quantified using Keyence BZ-X700 Microscope and accompanying Batch Analysis Software to determine the average area of positive signal (RNA) or the mean number of SHIV.D DNA-containing nuclei (DNA) from 10 nonoverlapping 20X images per brain section of each RM **(B)**. 20X frame area = 393,880 μm². Box and whisker plots are presented as mean values of counts ± quartiles, with whiskers representing the range

Viral DNA and RNAscope, a highly specific and sensitive next generation in situ hybridization (ISH) technique, was performed on FFPE occipital, temporal, and frontal cortex sections and quantified (Fig. 2). RNAscope and DNAscope can detect single virions and transcripts, and resulting RNA- and DNA-positive signal can be used to quantify viral nucleic acids and differentiate between active viral replication and low-level viral RNA transcription [33]. Punctate, nuclear RNA signal represents viral particles or low-level transcription of viral RNA, while large areas of high intensity signal amplification represent active viral replication [33]. Using RNAscope, high levels of viral RNA were found in animals in the progression group, with some localizing to vacuolar lesions. Lower levels of viral RNA persisted in the CNS in animals from the off-ART and on-ART cohorts. Using a probe targeting the sense strand of SHIV.D DNA, proviral DNA was detected inside the nuclei of cells in all three treatment groups (Fig. 2). Both replicating SHIV.D RNA and proviral DNA were found in the CNS of RM GA67 after 6 months of ART suppression. The mean SHIV.D RNA positive signal area in the progression group was 2.13 and 2368.7 µm^2^ for DE33 and EJ94, respectively. In the off-ART group, the mean area of RNA positive signal was 3.20 (FE43) and 33.3 µm^2^ (FT42). In the on-ART group, the mean area of the SIV RNA positive signal was 12.6 (FR55) and 21.3 µm^2^ (GA67). The mean number of SHIV.D DNA + nuclei in the progression group was 1.95 (DE33) and 13.7 cells/mm^2^ (EJ94). In the off-ART group, the mean number of SHIV.D DNA + cells was 1.35 (FE43) and 1.27 cells/mm^2^ (FT42). In the on-ART group, the number of viral DNA + cells was 2.37 (FR55) and 0.846 cells/mm^2^ (GA67).

### SHIV.D replicates in myeloid cells during ART suppression

**Fig. 3:**
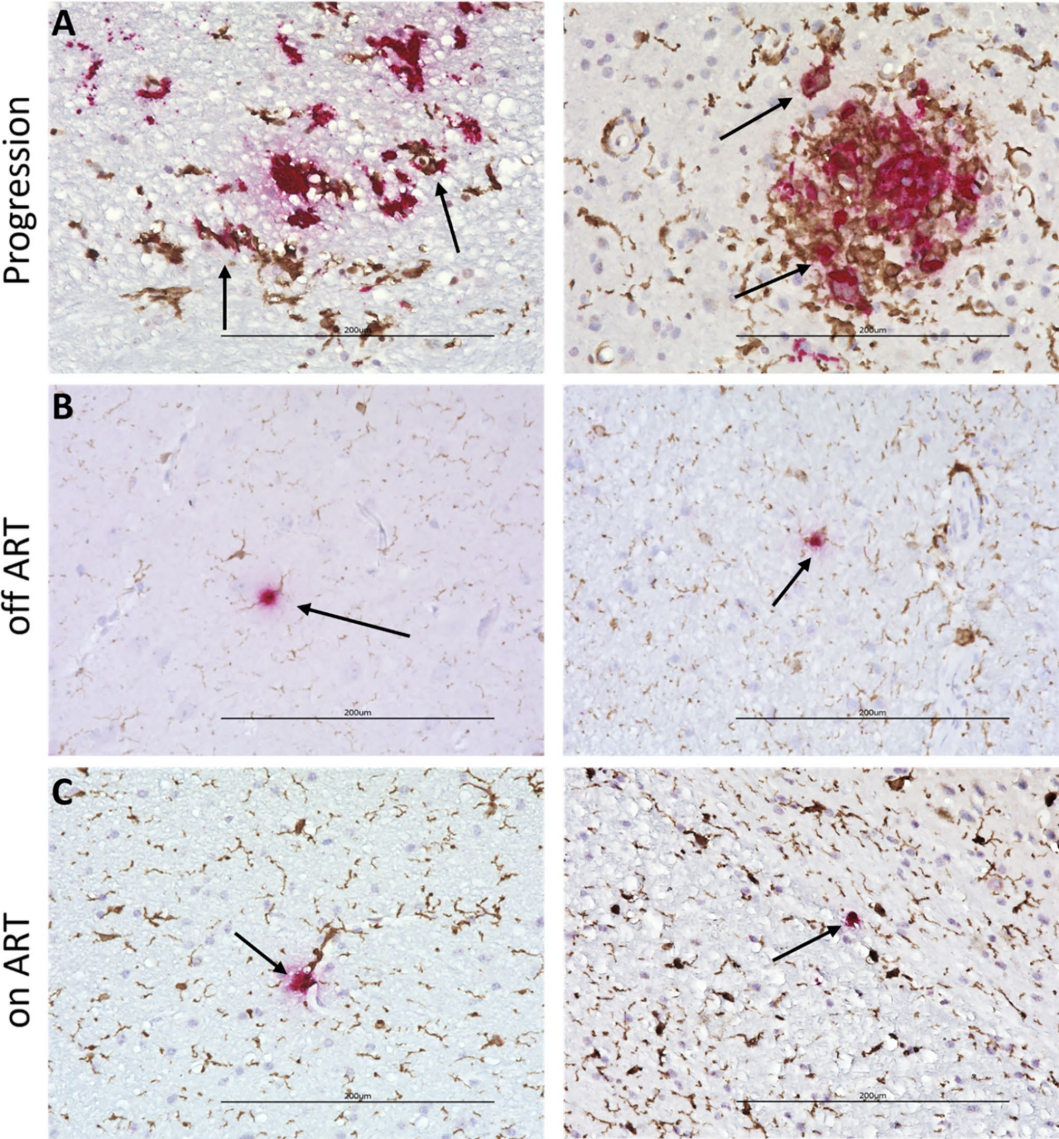
TF SHIV.D replicates and persists in myeloid cells. Dual RNAscope ISH (red) and IBA1 IHC (brown) reveal RNA/IBA1 + microglia/macrophage co-localization (arrows) in viremic RM EJ94 **(A)** and at low levels during ATI in off ART RM FE43 and FT42 **(B)** and ART suppression in on-ART RM GA67 and FR55 **(C).** Representative images are 40X magnification. Scale bar = 200 μm

Dual target IHC/RNAscope co-staining was performed on occipital and temporal cortex sections (Fig. 3). Colocalization between IBA1+ (brown) microglia and macrophages and SHIV.D RNA (red) was frequent and present in all three treatment groups, including RM GA67 after 6 months ART suppression (Fig. 3). In Progression RM (Fig. 3A), syncytia and clusters of microglial cells and SHIV.D + RNA were observed at the site of vacuolar lesions. Colocalization between CD3 + T cells and SHIV.D RNA was not prevalent (Supplemental Fig. 3). This demonstrates persistent SHIV.D viral replication inside CNS myeloid cells in the CNS during ART suppression.

## Discussion

A NHP model of HIV persistence in the CNS is critical to elucidate viral reservoir dynamics and studying viral pathogenesis and persistence in the brain. Because human brain samples are scarce and available only in rare clinical scenarios requiring biopsy or postmortem, NHP models are crucial to studying the progression of HIV-associated disease in the CNS. Most SIV/NHP models are associated with rapid progression of disease and onset of SIV encephalitis [6, 34]. Early SHIV/NHP models with less overt encephalitis failed to consistently maintain CD4 and CCR5 tropism, limiting their biological relevance in the CNS [25, 28, 35]. A body of work now demonstrates the relevance of SHIVs encoding TF HIV-1 envs, as they mirror viral kinetics, lymphoid pathogenesis, and induction of authentic HIV-specific adaptive immune responses [12, 28, 35, 36]. Previous research determined that TF SHIV.D.191,859, specifically, recapitulates additional components of HIV-1 infection, including ongoing viremia without frequent spontaneous control, and rapid suppression of viremia with ART with viral persistence in the periphery and lymphoid reservoirs [31]. Here, we demonstrate that TF SHIV.D actively replicates in the brain, causes neuropathogenesis, and persists in the CNS through antiretroviral therapy in rhesus macaques.

Using RNAscope in situ hybridization, we demonstrate that SHIV.D replicates in the CNS during viremia, but also at low levels during ATI and ART suppression. Large, high-intensity areas of RNAscope signal indicating active viral replication were identified in RM in all three treatment groups [33]. Though SHIV.D is dual-tropic and known to efficiently infect CD4 + T cells in vitro and systemically [25, 28], we found replicating virus predominantly in myeloid cells in the CNS. This is consistent with CNS HIV-1 infection in humans, where macrophages, microglia, and monocytes are the primary infected cell types in the brain [20, 24, 37–41]. Additionally, TF SHIV.D replication persists at low levels in the CNS despite undetectable plasma viremia. This emulates HIV-1 CNS pathogenesis in humans, potentially due to the inefficacy of ART drugs crossing the blood-brain barrier [42–44], and solidifies the need for robust CNS persistence research with a biologically relevant animal model.

Using DNAscope, we found proviral SHIV.D DNA in multiple brain regions in animals both on and off ART. Previous SHIVs and SIV/RM model systems have been unable to replicate continuous CCR5 tropism in RMs, limiting infection and pathogenesis in the brain [12, 25, 31, 32, 35, 45]. This further validates the potential for use of TF SHIV.D in latency and curative research in the CNS reservoir.

Using immunohistochemistry, we found myeloid-mediated inflammation, inflammatory foci, and vacuolar lesions and degeneration in the CNS of TF SHIV.D progression animals. Multinucleated giant cells and clusters of microglial cells at the site of lesions were also observed in some RM. Interestingly, lower levels of inflammation persisted in the off-ART RMs and ART-suppressed RMs even in the absence of viral RNA or DNA. Further research to investigate potential mechanisms of persistent inflammation not localized in areas of high levels of SHIV.D replication is in progress.

This study has several limitations. The sample size (n = 6, 2 per group) was small, which hindered statistical analyses. However, additional studies using this animal model to examine CNS pathogenesis are underway. The CNS samples were from a small retrospective study of banked brain tissues. Since we have demonstrated that TF SHIV.D is a biologically relevant model for HIV pathogenesis in the CNS, additional studies with larger RM cohorts are underway. Secondly, the number of available archived tissue samples was limited, and no fresh frozen brain tissue was available. Finally, cerebrospinal fluid (CSF) samples were not obtained in these animals, so no CSF viral loads could be obtained. CSF viral load levels would have been of particular interest for RM FT42, which spontaneously controlled plasma viremia, but which nonetheless maintained CNS viral replication. In ongoing studies, larger cohorts of RMs infected with TF SHIV.D virus will elucidate viral kinetics, reservoir dynamics, and mechanisms of persistent pathogenesis in the CNS. This research demonstrates that the TF SHIV.D/RM model is valuable for investigating the dynamics of HIV neuropathogenesis and persistence in the CNS reservoir.

## Methods

### Non-human primates

Archived tissue samples from 6 female rhesus macaques (RMs) were obtained from collaborators at the Tulane National Primate Research Center (TNPRC) and the University of Pennsylvania and described previously [31]. In the study from which RMs were retrospectively studied, the protocol was for animals to be challenged intravaginally, then if not productively infected after 4 attempts, to be challenged intravenously. Thus, RMs from the same protocol were infected vaginally (4) and intravenously (2). As described [28], plasma viral kinetics and reservoir dynamics were similar despite the challenge route. RMs were maintained at TNPRC according to the Association for Assessment and Accreditation of Laboratory Animal Care standards. All experiments were approved by the Tulane Animal Care and Use Committee. All macaques were negative for SIV controller alleles Mamu-A*01, B*08, and B*17 with the exception of DE33, which was A*01 positive [31]. Following perfusion and formalin fixation at necropsy, brain cortex lobes were isolated and embedded in paraffin blocks.

### Immunohistochemistry

Formalin-fixed paraffin-embedded (FFPE) tissue blocks of the occipital, temporal, and frontal lobes were obtained from Tulane National Primate Research Center (TNRPC). Tissue blocks were cut to 5 microns and placed on charged slides. Slides were deparaffinized in xylene for 2 × 10 min and rehydrated using an ethanol gradient for 10 min in each concentration (100%, 90%, 70%). Antigen retrieval was performed by heating slides in Antigen Unmasking Solution, citrate buffer (Vector) diluted in water at 92 °C for 20 min, then cooled to room temperature (RT) for 20 min. Following heat pre-treatment antigen retrieval, sections were washed in water (5 min), then PBST (0.05% Tween 20) for 5 min. Sections were then incubated with Bloxall Endogenous Peroxidase and Alkaline Phosphatase Blocking Solution (Vector) for 10 min in a humidity chamber at RT. After 2 × 5 min PBST wash, sections were incubated with Protein Block, serum-free (Dako) for 30 min at RT. Primary antibodies were diluted in antibody diluent (Dako) and incubated at 4 °C overnight (Supplemental Table 2). After 2 × 5 min PBST washes, sections were incubated with appropriate HRP conjugated secondary antibody (Dako) for 30 min in dark at RT (Supplemental Table 2). After 2 × 5 min PBST wash, antigens were detected by DAB chromogen development (1 drop DAB chromogen per 1mL DAB + substrate buffer (Dako)) and counterstained with hematoxylin for 30 s. Sections were dehydrated using an ethanol gradient for 2 × 2 min each concentration (70%, 90%, 100%) and 2 × 5 min xylene. Coverslips were mounted using permanent mounting media. Slides were imaged using Keyence BZ-X700 microscope and positively stained cell counts were quantified using accompanying Batch Analysis Software. Cell count was quantified using Batch Analysis Software and averaged from 10 nonoverlapping 20X images per brain section of each RM (Fig. 1). Total average values for each cell type in each RM were calculated by taking the arithmetic mean from all representative images from all three brain sections studied (10 per brain region).

### In situ hybridization

SHIV.D RNA and DNA were visualized using RNAscope and DNAscope in situ hybridization according to the specifications of the manufacturer (ACD Bio). The probe utilized for RNAscope (ACD) was a SIVmac anti-sense RNA probe. RNAscope probes do not bind to double-stranded DNA and only bind to single-stranded anti-sense RNA. Formalin-fixed paraffin-embedded (FFPE) tissue blocks of the occipital, temporal, and frontal lobes were obtained from Tulane National Primate Research Center (TNRPC). Tissue blocks were cut to 5 microns and placed on charged slides. Slides were deparaffinized in xylene for 10 min, washed 2 × 2 min in 100% ethanol, and air dried. Sections were boiled for 15 min in 1X Target Retrieval solution (ACD Bio) and removed to water. Tissue sections were treated with hydrogen peroxide for 10 min at RT, then protease for 30 min in a humidity chamber at 40 °C. Custom probes targeting SHIV.D RNA and DNA (sense strand) were designed with ACD Bio. RNA and DNA probes were hybridized for 2 h in a humidity chamber at 40 °C. RNA and DNA were detected by amplification and chromogenic development using RNAscope 2.5 High Definition – Red Assay kit (ACD Bio). Tissue sections were counterstained with hematoxylin for 30 s, washed in water, then dried in a 60° dry oven for 15 min. Slides were cleaned with xylene and coverslips were mounted using permanent mounting media. Slides were imaged using Keyence BZ-X700 microscope and the RNA area was quantified using accompanying Batch Analysis Software. The area of positive signal (RNA) or the number of nuclei containing positive signal (DNA) was quantified using Batch Analysis Software and averaged from 10 nonoverlapping 20X images per brain section of each RM (Fig. 2). Total average values for each nucleic acid in each RM were calculated by taking the arithmetic mean from all representative images from all three brain sections studied (10 per brain region).

### Dual target IHC/ISH

FFPE brain tissue section slides were deparaffinized in xylene for 2 × 10 min, rehydrated using an ethanol gradient for 2 × 2 min in each concentration (100%, 90%, 70%), then washed in water. Antigen retrieval was performed by heating slides in Antigen Unmasking Solution, citrate buffer (Vector) diluted in water at 92 °C for 20 min, then cooled to room temperature (RT) for 20 min. Following heat pre-treatment antigen retrieval, sections were washed in water (5 min), then PBST (0.05% Tween 20) for 5 min. Sections were then incubated with Bloxall Endogenous Peroxidase and Alkaline Phosphatase Blocking Solution (Vector) for 10 min in a humidity chamber at RT. After 2 × 5 min wash with 1X RNAscope Wash Buffer (ACD Bio), RNA probes were hybridized for 2 h in humidity chamber at 40 °C. RNA amplification was performed using RNAscope 2.5 High Definition – Red Assay kit (ACD Bio) according to the specifications of the manufacturer. After 2 × 5 min PBST wash, sections were incubated with Protein Block, serum-free (Dako) for 30 min at RT. Primary antibodies were diluted in Antibody diluent (Dako) at 4 °C overnight. After 3 × 10 min PBST wash, sections were incubated with appropriate HRP conjugated secondary antibody (Dako) for 30 min in dark at RT. After 2 × 5 min PBST wash, antigens were detected by DAB chromogen development (1 drop DAB chromogen per 1mL DAB + substrate buffer [Dako]) and counterstained with hematoxylin for 30 s. Slides were washed in water and dried in 60 °C dry oven for 15 min. Slides were cleaned with xylene and coverslips were mounted using permanent mounting media. Slides were imaged using Keyence BZ-X700 microscope.

### Analyses

All graphs were generated using GraphPad Prism 8.2 software.

### Electronic supplementary material

Below is the link to the electronic supplementary material.

Supplementary Material 1

## Data Availability

Data and reagents available upon a reasonable request to the corresponding authors.

## References

[1] Castro-Gonzalez S, Colomer-Lluch M, Serra-Moreno R (2018). Barriers for HIV cure: the Latent Reservoir. AIDS Res Hum Retroviruses.

[2] Koenig S, Gendelman HE, Orenstein JM, Canto MCD, Pezeshkpour GH, Yungbluth M, Janotta F, Aksamit A, Martin MA, Fauci AS. Detection of AIDS Virus in Macrophages in Brain tissue from AIDS patients with Encephalopathy. Science; 1986. 10.1126/science.3016903 3016903

[3] Coleman CM, Wu L (2009). HIV interactions with monocytes and dendritic cells: viral latency and reservoirs. Retrovirology.

[4] Kumar A, Abbas W, Herbein G (2014). HIV-1 latency in Monocytes/Macrophages. Viruses.

[5] Moretti S, Virtuoso S, Sernicola L, Farcomeni S, Maggiorella MT, Borsetti A. Advances in SIV/SHIV Non-Human Primate Models of NeuroAIDS. Pathogens 2021, 10(8). 10.3390/pathogens10081018 PMC8398602 34451482

[6] Obregon-Perko V, Bricker K, Chahroudi A (2020). The Brain retains: Nonhuman Primate Models for Pediatric HIV-1 in the CNS. Curr HIV/AIDS Rep.

[7] Raghavan R, Stephens EB, Joag SV, Adany I, Pinson DM, Li Z, Jia F, Sahni M, Wang C, Leung K, Foresman L, Narayan O (2008). Neuropathogenesis of chimeric Simian/Human immunodeficiency virus infection in Pig-tailed and Rhesus Macaques. Brain Pathol.

[8] Hsu DC, Sunyakumthorn P, Wegner M, Schuetz A, Silsorn D, Estes JD, Deleage C, Tomusange K, Lakhashe SK, Ruprecht RM, Lombardini E, Im-Erbsin R, Kuncharin Y, Phuang-Ngern Y, Inthawong D, Chuenarom W, Burke R, Robb ML, Ndhlovu LC, Ananworanich J, Valcour V, O’Connell RJ, Spudich S, Michael NL, Vasan S. Central nervous system inflammation and infection during early, nonaccelerated simian-human immunodeficiency virus infection in Rhesus Macaques. J Virol 2018, 92(11). 10.1128/JVI.00222-18 PMC5952152 29563297

[9] Humbert M, Rasmussen RA, Song R, Ong H, Sharma P, Chenine AL, Kramer VG, Siddappa NB, Xu W, Else JG, Novembre FJ, Strobert E, O’Neil SP, Ruprecht RM (2008). SHIV-1157i and passaged progeny viruses encoding R5 HIV-1 clade C env cause AIDS in rhesus monkeys. Retrovirology.

[10] Zhuang K, Leda AR, Tsai L, Knight H, Harbison C, Gettie A, Blanchard J, Westmoreland S, Cheng-Mayer C (2014). Emergence of CD4 independence envelopes and astrocyte infection in R5 simian-human immunodeficiency Virus Model of Encephalitis. J Virol.

[11] Buch S, Sui Y, Dhillon N, Potula R, Zien C, Pinson D, Li S, Dhillon S, Nicolay B, Sidelnik A, Li C, Villinger T, Bisarriya K, Narayan O (2004). Investigations on four host response factors whose expression is enhanced in X4 SHIV encephalitis. J Neuroimmunol.

[12] Bar KJ, Coronado E, Hensley-McBain T, O’Connor MA, Osborn JM, Miller C, Gott TM, Wangari S, Iwayama N, Ahrens CY, Smedley J, Moats C, Lynch RM, Haddad EK, Haigwood NL, Fuller DH, Shaw GM, Klatt NR, Manuzak JA. Simian-Human Immunodeficiency Virus SHIV.CH505 Infection of Rhesus Macaques Results in Persistent Viral Replication and Induces Intestinal Immunopathology. J Virol 2019, 93(18). 10.1128/JVI.00372-19 PMC6714786 31217249

[13] Barton K, Winckelmann A, Palmer S (2016). HIV-1 Reservoirs during suppressive therapy. Trends Microbiol.

[14] Chan CN, Trinité B, Lee CS, Mahajan S, Anand A, Wodarz D, Sabbaj S, Bansal A, Goepfert PA, Levy DN. HIV-1 latency and virus production from unintegrated genomes following direct infection of resting CD4 T cells. Retrovirology 2016, 13. 10.1186/s12977-015-0234-9 PMC4700562 26728316

[15] Finzi D, Hermankova M, Pierson T, Carruth LM, Buck C, Chaisson RE, Quinn TC, Chadwick K, Margolick J, Brookmeyer R, Gallant J, Markowitz M, Ho DD, Richman DD, Siliciano RF. Identification of a Reservoir for HIV-1 in patients on highly active antiretroviral therapy. Science; 1997. 10.1126/science.278.5341.1295 9360927

[16] Pinkevych M, Cromer D, Tolstrup M, Grimm AJ, Cooper DA, Lewin SR, Søgaard OS, Rasmussen TA, Kent SJ, Kelleher AD, Davenport MP (2015). HIV Reactivation from latency after treatment interruption occurs on average every 5–8 days—implications for HIV Remission. PLoS Pathog.

[17] Bednar MM, Sturdevant CB, Tompkins LA, Arrildt KT, Dukhovlinova E, Kincer LP, Swanstrom R (2015). Compartmentalization, viral evolution, and viral latency of HIV in the CNS. Curr HIV/AIDS Rep.

[18] Sonti S, Sharma AL, Tyagi M (2021). HIV-1 persistence in the CNS: mechanisms of latency, pathogenesis and an update on eradication strategies. Virus Res.

[19] Balcom EF, Roda WC, Cohen EA, Li MY, Power C (2019). HIV-1 persistence in the central nervous system: viral and host determinants during antiretroviral therapy. Curr Opin Virol.

[20] Wallet C, De Rovere M, Van Assche J, Daouad F, De Wit S, Gautier V, Mallon PWG, Marcello A, Van Lint C, Rohr O, Schwartz C (2019). Microglial cells: the Main HIV-1 Reservoir in the brain. Front Cell Infect Microbiol.

[21] Trease AJ, Niu M, Morsey B, Guda C, Byrareddy SN, Buch S, Fox HS. Antiretroviral therapy restores the homeostatic state of microglia in SIV-infected rhesus macaques. J Leukoc Biol 2022. 10.1002/JLB.3HI0422-635R PMC9796061 35686500

[22] Schnell G, Spudich S, Harrington P, Price RW, Swanstrom R (2009). Compartmentalized human immunodeficiency virus type 1 originates from long-lived cells in some subjects with HIV-1-associated dementia. PLoS Pathog.

[23] Veenstra M, León-Rivera R, Li M, Gama L, Clements JE, Berman JW. Mechanisms of CNS Viral Seeding by HIV + CD14 + CD16 + Monocytes: Establishment and Reseeding of Viral Reservoirs Contributing to HIV-Associated Neurocognitive Disorders. mBio 2017, 8(5). 10.1128/mBio.01280-17 PMC5654927 29066542

[24] Ko A, Kang G, Hattler JB, Galadima HI, Zhang J, Li Q, Kim W (2019). Macrophages but not astrocytes Harbor HIV DNA in the brains of HIV-1-Infected aviremic individuals on suppressive antiretroviral therapy. J Neuroimmune Pharmacol.

[25] Bauer AM, Bar KJ (2020). Advances in simian–human immunodeficiency viruses for nonhuman primate studies of HIV prevention and cure. Curr Opin HIV AIDS.

[26] Joseph SB, Swanstrom R, Kashuba ADM, Cohen MS (2015). Bottlenecks in HIV-1 transmission: insights from the study of founder viruses. Nat Rev Microbiol.

[27] Baalwa J, Wang S, Parrish NF, Decker JM, Keele BF, Learn GH, Yue L, Ruzagira E, Ssemwanga D, Kamali A, Amornkul PN, Price MA, Kappes JC, Karita E, Kaleebu P, Sanders E, Gilmour J, Allen S, Hunter E, Montefiori DC, Haynes BF, Cormier E, Hahn BH, Shaw GM (2013). Molecular identification, cloning and characterization of transmitted/founder HIV-1 subtype A, D and A/D infectious molecular clones. Virology.

[28] Li H, Wang S, Kong R, Ding W, Lee F, Parker Z, Kim E, Learn GH, Hahn P, Policicchio B, Brocca-Cofano E, Deleage C, Hao X, Chuang G, Gorman J, Gardner M, Lewis MG, Hatziioannou T, Santra S, Apetrei C, Pandrea I, Alam SM, Liao H, Shen X, Tomaras GD, Farzan M, Chertova E, Keele BF, Estes JD, Lifson JD, Doms RW, Montefiori DC, Haynes BF, Sodroski JG, Kwong PD, Hahn BH, Shaw GM (2016). Envelope residue 375 substitutions in simian-human immunodeficiency viruses enhance CD4 binding and replication in rhesus macaques. Proc Natl Acad Sci U S A.

[29] Sacktor N, Nakasujja N, Skolasky RL, Rezapour M, Robertson K, Musisi S, Katabira E, Ronald A, Clifford DB, Laeyendecker O, Quinn TC (2009). HIV Subtype D is Associated with Dementia, compared with subtype A, in immunosuppressed individuals at risk of cognitive impairment in Kampala, Uganda. Clin Infect Dis.

[30] Tyor W, Fritz-French C, Nath A (2013). Effect of HIV clade differences on the onset and severity of HIV-associated neurocognitive disorders. J Neurovirol.

[31] Bauer AM, Ziani W, Lindemuth E, Kuri-Cervantes L, Li H, Lee F, Watkins M, Ding W, Xu H, Veazey R, Bar KJ. Novel Transmitted/Founder simian-human immunodeficiency viruses for human immunodeficiency virus latency and Cure Research. J Virol 2020, 94(8). 10.1128/JVI.01659-19 PMC7108852 31969435

[32] Li H, Wang S, Kong R, Ding W, Lee F, Parker Z, Kim E, Learn GH, Hahn P, Policicchio B, Brocca-Cofano E, Deleage C, Hao X, Chuang G, Gorman J, Gardner M, Lewis MG, Hatziioannou T, Santra S, Apetrei C, Pandrea I, Alam SM, Liao H, Shen X, Tomaras GD, Farzan M, Chertova E, Keele BF, Estes JD, Lifson JD, Doms RW, Montefiori DC, Haynes BF, Sodroski JG, Kwong PD, Hahn BH, Shaw GM (2016). Envelope residue 375 substitutions in simian–human immunodeficiency viruses enhance CD4 binding and replication in rhesus macaques. Proc Natl Acad Sci U S A.

[33] Deleage C, Chan CN, Busman-Sahay K, Estes JD. Next-generation in situ hybridization approaches to define and quantify HIV and SIV reservoirs in tissue microenvironments. Retrovirology 2018, 15(4). 10.1186/s12977-017-0387-9 PMC5761108 29316956

[34] Lee CA, Beasley E, Sundar K, Smelkinson M, Vinton C, Deleage C, Matsuda K, Wu F, Estes JD, Lafont BAP, Brenchley JM, Hirsch VM. Simian Immunodeficiency Virus-Infected Memory CD4 + T Cells Infiltrate to the Site of Infected Macrophages in the Neuroparenchyma of a Chronic Macaque Model of Neurological Complications of AIDS. mBio 2020, 11(2). 10.1128/mBio.00602-20 PMC7175093 32317323

[35] Li H, Wang S, Lee F, Roark RS, Murphy AI, Smith J, Zhao C, Rando J, Chohan N, Ding Y, Kim E, Lindemuth E, Bar KJ, Pandrea I, Apetrei C, Keele BF, Lifson JD, Lewis MG, Denny TN, Haynes BF, Hahn BH, Shaw GM. New SHIVs and Improved Design Strategy for modeling HIV-1 transmission, immunopathogenesis, Prevention and Cure. J Virol 2021, 95(11). 10.1128/JVI.00071-21 PMC8139694 33658341

[36] Roark RS, Li H, Williams WB, Chug H, Mason RD, Gorman J, Wang S, Lee F, Rando J, Bonsignori M, Hwang K, Saunders KO, Wiehe K, Moody MA, Hraber PT, Wagh K, Giorgi EE, Russell RM, Bibollet-Ruche F, Liu W, Connell J, Smith AG, DeVoto J, Murphy AI, Smith J, Ding W, Zhao C, Chohan N, Okumura M, Rosario C, Ding Y, Lindemuth E, Bauer AM, Bar KJ, Ambrozak D, Chao CW, Chuang G, Geng H, Lin BC, Louder MK, Nguyen R, Zhang B, Lewis MG, Raymond DD, Doria-Rose NA, Schramm CA, Douek DC, Roederer M, Kepler TB, Kelsoe G, Mascola JR, Kwong PD, Korber BT, Harrison SC, Haynes BF, Hahn BH, Shaw GM. Recapitulation of HIV-1 env-antibody coevolution in macaques leading to neutralization breadth. Science 2021, 371(6525). 10.1126/science.abd2638 PMC8040783 33214287

[37] Cenker JJ, Stultz RD, McDonald D (2017). Brain microglial cells are highly susceptible to HIV-1 infection and spread. AIDS Res Hum Retroviruses.

[38] Borrajo A, Spuch C, Penedo MA, Olivares JM, Agís-Balboa RC (2021). Important role of microglia in HIV-1 associated neurocognitive disorders and the molecular pathways implicated in its pathogenesis. Ann Med.

[39] Burdo TH: Editor’s Commentary for Special Issue, editor. “The role of macrophages in HIV persistence”. J Neuroimmune Pharmacol 2019, 14(1):2–5. 10.1007/s11481-019-09836-3 PMC6424350 30737724

[40] Burdo TH, Lackner A, Williams KC (2013). Monocyte/macrophages and their role in HIV neuropathogenesis. Immunol Rev.

[41] Llewellyn GN, Alvarez-Carbonell D, Chateau M, Karn J, Cannon PM (2018). HIV-1 infection of microglial cells in a reconstituted humanized mouse model and identification of compounds that selectively reverse HIV latency. J Neurovirol.

[42] Zhang Y, Ouyang Y, Liu L, Chen D (2015). Blood-brain barrier and neuro-AIDS. Eur Rev Med Pharmacol Sci.

[43] Gisslén M, Price RW, Nilsson S (2011). The definition of HIV-associated neurocognitive disorders: are we overestimating the real prevalence?. BMC Infect Dis.

[44] Bhaskaran K, Mussini C, Antinori A, Walker AS, Dorrucci M, Sabin C, Phillips A, Porter K (2008). Changes in the incidence and predictors of human immunodeficiency virus-associated dementia in the era of highly active antiretroviral therapy. Ann Neurol.

[45] Finzi A, Pacheco B, Xiang S, Pancera M, Herschhorn A, Wang L, Zeng X, Desormeaux A, Kwong PD, Sodroski J (2012). Lineage-specific differences between human and simian immunodeficiency virus regulation of gp120 trimer association and CD4 binding. J Virol.

