# Supplementary material for: Transmitted/founder SHIV.D replicates in the brain, causes neuropathogenesis, and persists on combination antiretroviral therapy in rhesus macaques
Source: Retrovirology. 2023 Aug 10;20:13. doi: 10.1186/s12977-023-00628-5 (PMC10413509; doi:10.1186/s12977-023-00628-5)
Supplement: Supplementary file 1 — Supplementary Material 1 [file 12977_2023_628_MOESM1_ESM.docx]

**Supplementary Materials**

**Transmitted/founder SHIV.D replicates in the brain, causes neuropathogenesis, and persists on combination antiretroviral therapy in rhesus macaques**

Rachel M. Podgorski^1^, Jake A. Robinson^1^, Mandy D. Smith^1^, Suvadip Mallick^2^, Huaqing Zhao^3^, Ronald S. Veazey^4^, Dennis L. Kolson^5^, Katharine J. Bar^2^*, and Tricia H. Burdo^1^*

^1^Center for NeuroVirology and Gene Editing, Department of Microbiology, Immunology, and Inflammation, Lewis Katz School of Medicine, Temple University, Philadelphia, PA, USA.

^2^Department of Medicine, Perelman School of Medicine, University of Pennsylvania, Philadelphia, PA, USA.

^3^Center for Biostatistics and Epidemiology, Department of Biomedical Education and Data Science, Lewis Katz School of Medicine, Temple University, Philadelphia, PA, USA.

Department of Neurology, Perelman School of Medicine, University of Pennsylvania, Philadelphia, PA, USA.

^4^Tulane National Primate Research Center, Tulane School of Medicine, Covington, LA, USA.

^5^Department of Neurology, Perelman School of Medicine, University of Pennsylvania, Philadelphia, PA, USA.

*Corresponding Authors: Katharine J. Bar, University of Pennsylvania (bark@pennmedicine.upenn.edu) and Tricia H. Burdo, Temple University ([burdot@temple.edu](mailto:burdot@temple.edu))

| **RM** | **Treatment group** | **Mode of infection** | **Plasma VL at necropsy** | **Months post infection** | **Reason for euthanasia** | **Notable CNS pathology** | **Notable peripheral pathology** |
| --- | --- | --- | --- | --- | --- | --- | --- |
| DE33 | Progression | intravaginal | 10^6^ c/mL | 18 | clinical deterioration | Nothing of significance. | Lymphoid hyperplasia of bone marrow [3-4]. Chronic erosive inflammation of ileocecal valve [3]. Lymphoid hyperplasia of pancreas [2]. Lymphoid hyperplasia of tonsil, lymph node [4]. Nodular hyperplasia of kidney [3]. Cytoplasmic vacuolization of hepatic parenchymal cells [4]. Nodular hyperplasia of lymphoid tissue of lung [1]. |
| EJ94 | Progression | intravaginal | 10^7^ c/mL | 39 | clinical deterioration | Vacuolar degeneration, vacuolar lesions with few lymphocytes. | Chronic inflammation of lung with edema and epithelial hyperplasia [4]. Syncytial giant cells in lung [3]. Chronic inflammation of liver [1]. Diffuse lymphoid hyperplasia of lymph node [2]. Severe endometriosis [4]. |
| FE43 | off ART (ATI) | intravaginal | 10^4^ c/mL | 40 | tissue collection | Multiple small inflammatory foci in occipital lobe, subcortical white matter, and meninges [1]. | Chronic inflammation of lung, liver, and interstitial tissue of kidney [1]. Diffuse lymphoid hyperplasia of lymph node and splenic lymphatic follicle [2-3]. |
| FT42 | off ART (ATI) | intravaginal | <83 c/mL | 36 | tissue collection | Predominately perivascular inflammation of frontal cortex, temporal lobe, and thalamus [1]. | Perivascular inflammation of lung [2]. Chronic inflammation of stomach [3]. Perivascular inflammation of ileum and cecum [1]. |
| FR55 | on ART | intravenous | <83 c/mL | 45 | clinical deterioration | Nothing of significance. | Focal granulomatous inflammation and histiocytosis of lymph node [2]. Mycobacterium avium mediated inflammation and necrosis of lymph node [4]. M. avium mediated granulomatous inflammation of stomach, jejunum, and colon [2-3]. |
| GA67 | on ART | intravenous | <83 c/mL | 35 | tissue collection | Nothing of significance. | Nothing of significance. |

**Supplemental Table 1. TF** **SHIV.D infection and pathological findings at necropsy.** Pathology finding determination and grading performed by a TNPRC veterinary pathologist. Pathology grading degrees: [1] = minimal; [2] = mild; [3] = moderate; [4] = severe.

| Primary antibody | Source | Concentration | Antigen retrieval concentration | Secondary antibody | Source |
| --- | --- | --- | --- | --- | --- |
| CD3 | Agilent (A0452) | 1:800 | 1:100 | HRP labelled Anti-Rabbit | Dako EnVision+ system (K4003) |
| CD68 | Agilent (M0814) | 1:400 | 1:200 | HRP labelled Anti-Mouse | Dako EnVision+ system (K4001) |
| MAC387 | Agilent (M0747) | 1:100 | None | HRP labelled Anti-Mouse | Dako EnVision+ system (K4001) |
| IBA1 | Millipore Sigma (MABN92) | 1:500 | 1:100 | HRP labelled Anti-Mouse | Dako EnVision+ system (K4001) |

**Supplemental Table 2. Immunohistochemistry antibodies and specifications.**

**
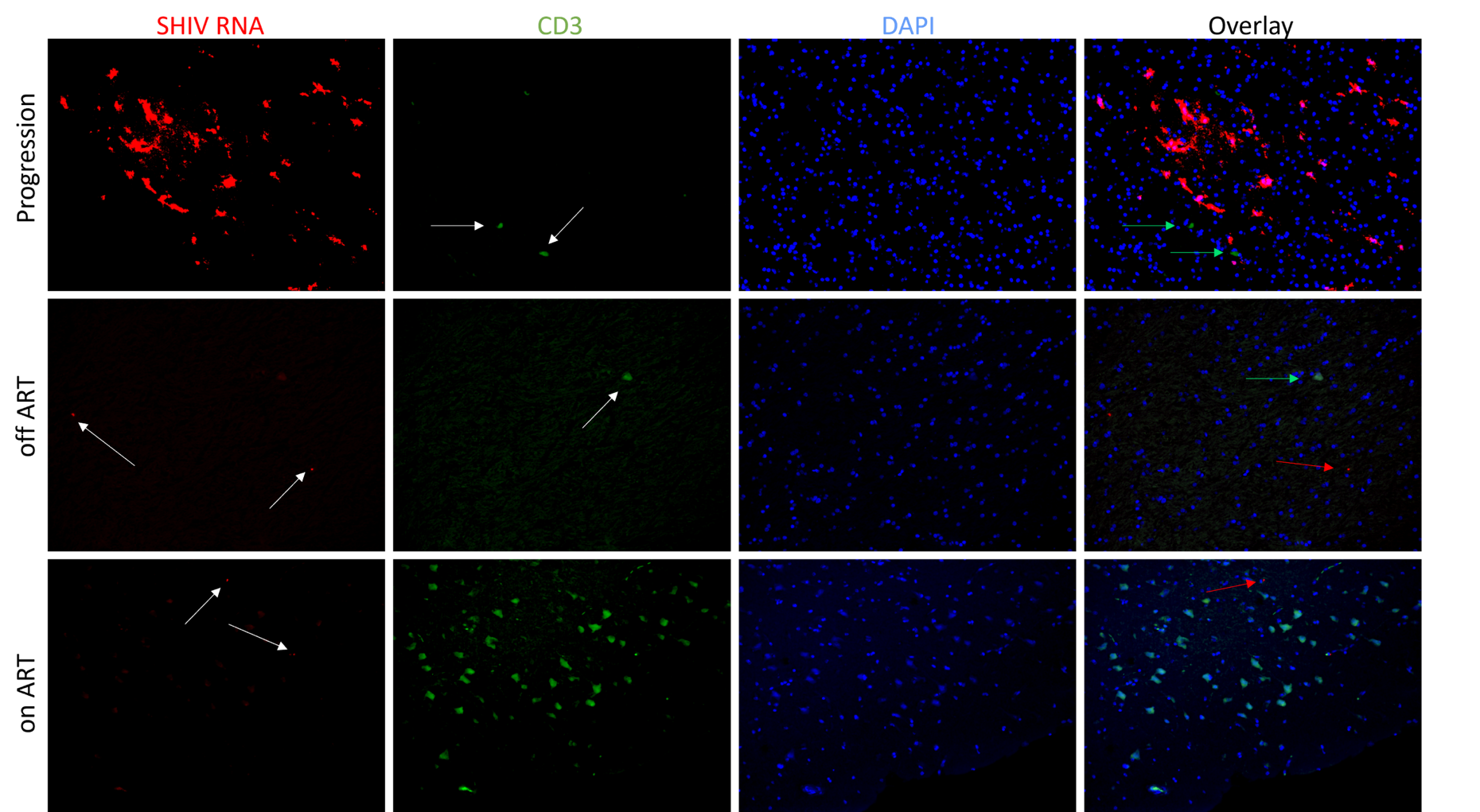
Supplemental Figure 3.** CD3/RNAscope Immunohistochemistry Overlay. Opal (Akoya) multiplex immunohistochemistry for CD3 (green), SHIV.D RNA (red), and DAPI (blue) targets was performed on brain tissue sections from Progression, off ART, and on ART RM. Co-localization between SHIV.D RNA and CD3+ was rarely observed.
